# Catching’s Compendium of Practical Dentistry

**Published:** 1897-05

**Authors:** 


### Catching's Compendium of Practical Dentistry.

The seventh volume of this valuable book has been issued for the year 1896. This work, under the careful preparation of Dr. B. H. Catching, has brought into a small and practical compass a very large amount of valuable information, gathered mainly from the journalistic literature of the world for the year 1896. The matter is classified and arranged into departments, rendering it very convenient for reference. The first division embraces Operative Dentistry; the second, Prosthetic Dentistry; the third, Crown and Bridge Work; fourth, Orthodentia; fifth, Dental Medicine; sixth, Oral Surgery; seventh, Miscellaneous; and eighth, Science in Dentistry, making the work very complete in its systematic arrangement. Dr. Catching has been at work in this direction for the last eight or ten years, and has attained great proficiency in the arrangement of his work. This is a work that should be in the hands of every dentist, and especially ot every one who wishes to keep abreast with the progress of the day. The work can be had directly from Dr. Catching, or through any of the dental dealers; indeed, almost any of the booksellers can procure it.

